# On Chlorotic Affections

**Published:** 1800-02

**Authors:** R. Scott

**Affiliations:** Newport, Isle of Wight


### *To the Editors of the Medical and Phyfical Journal.*

GENTLEMEN,

THE following remarks were occafioned by a paragraph in your number for September; and are an extract from a letter which I lately received from a much efteemed friend, who, though not a profeffional man, confiders medicine as not the leaft important branch of thofe general philofophical purfuits, which form the chief amufements of his leifure hours. You will, perhaps, think them of importance enough, to merit infertion in your very ufeful Journal. I am,

GENTLEMEN,

Your moft obedient fervant,

R. SCOTT, M.D.

*Newport, Ifle of Wight,*
*Dec.* 26, 1799.

---

"I was very much pleafed with the obfervations of Dr. Chifholm, as given in the number of the Medical Journal which you fent to me, and with the application by the Editors, of his opinion refpecting the *Cachexia Africana* to that very remarkable difeafe with which fo many females in civilized life are at a certain age affected. I have for fome time entertained a fimilar notion of the nature of that difeafe, but wanting opportunities of obfervation, and the means of inquiry, I had never thought of it farther than as a mere furmife of the fancy. It, indeed, appears to me to be by no means a new fuppofition, but to have occurred to many Pathologifts who have attentively confidered the difeafe, efpecially to thofe two eminent Nofologifts, Sauvages and Cullen. As probable caufes of their deferting it, and of their recurring to other, and, in my humble opinion, much lefs plaufible hypothefes, we may affign a dread of out-ftepping their own particular province, and perhaps too a wifh to keep up the dignity of man, and a fear of propagating an opinion which might, in improper hands, be made prejudicial to morality and the purity of focial life.

"In a philofophical view, however, the fexual paffion cannot be overlooked; and all muft acknowledge its influence, even when degraded to mere fenfualifm, and ftripped of that irrefiftible attraction which the neceffary reftraints and the general refinement of civilized life give to it. You are not to think that the attributing this difeafe to ungratified paffion, fuppofes the other fex to be more fubjugated to animal defires than ours. Although, indeed, ſuch an opinion might be ſaid to prove the inferiority of women in one reſpect, it would ſtill add to their character of chaſtity, and ſhew how much ſuperior they are to us in point of continence and ſelf-reſtraint. But far higher of the ſex I deem, than to imagine that in any view, whether of being more under the influence of paſſion, or in point of intellectual endowments, they are inferior to us; except you eſteem as a mark of inferiority what adds ſo much to their charms, that high ſuſceptibility of feeling which they generally poſſeſs, and which, in part, proceeds from the greater delicacy of bodily ſtructure, but chiefly, as I apprehend, from their peculiar education and habits in civilized life.

" I ſhould maintain that the male is, from having the ſame feelings and paſſions, equally liable as the female to this or a ſimilar diſeaſe (there neceſſarily ariſing a difference of bodily functions); and that we are to ſeek for the cauſe of its being in a manner confined to the latter in the very different circumſtances in which each ſex is placed.

" At the age at which this moſt powerful of all the paſſions begins to exert its influence on our frame, we are juſt entering on the buſy ſtage of life; where the great difference of ſcenery from what we have hitherto been accuſtomed to, the ardour we generally ſet out with, the deſire of diſtinguiſhing ourſelves, the freedom from reſtraint we now feel, and the difficulties we ſoon encounter, totally abſorb for the moſt part our mind, and prevent it from indulging in thoſe reveries, which when otherways unemployed, our imagination, conſtantly in action, is always forming.

" But the caſe of the female is widely different. Being in general endowed by Nature with keen feelings and a warm fancy, but, above all, having received from her preceptors and others high notions of the extreme delicacy and circumſpection required of female deportment, ſhe ſees herſelf placed upon a pinnacle, where ſhe is viewed by all around her with the moſt ſcrutinizing eye, and from which the leaſt lapſe of conduct would precipitate her into utter contempt. She cannot enter into thoſe active duties, that engage the attention of the male, and render him, if prudent, independent. She is precluded from being ever the firſt in thoſe advances, which lead to that tender and laſting union of the ſexes, to which we are ſo ſtrongly prompted by Nature, and which conſtitutes ſo great a ſhare of our happineſs.

[ To be continued in our next Number. ]

STATE

